# AutoRELACS: automated generation and analysis of ultra-parallel ChIP-seq

**DOI:** 10.1038/s41598-020-69443-8

**Published:** 2020-07-24

**Authors:** L. Arrigoni, F. Ferrari, J. Weller, C. Bella, U. Bönisch, T. Manke

**Affiliations:** 1 Max Planck Institute of Immunobiology and Epigenetics, Freiburg, Germany; 2 Faculty of Biology, University of Freiburg, Freiburg, Germany

**Keywords:** Chromatin immunoprecipitation, Next-generation sequencing, Bioinformatics, Chromatin analysis, Histone analysis

## Abstract

Chromatin immunoprecipitation followed by sequencing (ChIP-seq) is a method used to profile protein-DNA interactions genome-wide. Restriction Enzyme-based Labeling of Chromatin in Situ (RELACS) is a recently developed ChIP-seq protocol that deploys a chromatin barcoding strategy to enable standardized and high-throughput generation of ChIP-seq data. The manual implementation of RELACS is constrained by human processivity in both data generation and data analysis. To overcome these limitations, we have developed AutoRELACS, an automated implementation of the RELACS protocol using the liquid handler Biomek i7 workstation. We match the unprecedented processivity in data generation allowed by AutoRELACS with the automated computation pipelines offered by snakePipes. In doing so, we build a continuous workflow that streamlines epigenetic profiling, from sample collection to biological interpretation. Here, we show that AutoRELACS successfully automates chromatin barcode integration, and is able to generate high-quality ChIP-seq data comparable with the standards of the manual protocol, also for limited amounts of biological samples.

## Introduction

Chromatin immunoprecipitation followed by sequencing (ChIP-seq) is a widely used method to study protein-DNA interactions genome-wide^1^. Despite the enormous contribution that ChIP-seq has brought to our understanding of epigenetic and transcriptional control, the traditional ChIP-seq protocol^2,3^ presents various limitations. For example, it requires substantial amounts of biological input material, which is often a limiting factor in relevant clinical settings, and it is low-throughput, which prevents comprehensive epigenetic profiling. Furthermore, the protocol is poorly standardized across cell types, resulting in a high degree of technical variability that hampers biological interpretation of the data.

Over the last ten years, much work has been devoted to address these and other shortcomings^4–8^. In line with these efforts, we have recently developed Restriction Enzyme-based Labeling of Chromatin in Situ (RELACS), a method that employs chromatin barcoding to enable high-throughput generation of ChIP-seq experiments^9^. RELACS works reliably with low input material and can be used for quantitative ChIP-seq analysis^9,10^. The method is highly standardized, and could potentially be scaled to profile hundreds of samples in parallel for tens of DNA-binding proteins at once. Yet, the current manual implementation is limited by human processivity in both data generation and data analysis.

To match the ideal potential of this methodology, we have implemented an automated version of the RELACS protocol, named AutoRELACS, using the liquid handler Biomek i7 automated workstation (Beckman Coulter). While other automated ChIP-seq implementations already exist^11,12^, they still require a large amount of sample material, and they do not utilize the enormous multiplexing potential of barcoded chromatin. The scope of these methods is limited to data generation and the lack of an integrated bioinformatics workflow that streamlines standard computational tasks (e.g. QC, DNA-mapping, peak calling). AutoRELACS, on the other hand, couples the high-throughput generation of ChIP-seq data with the scalable and modular computational pipelines offered by snakePipes^13^. From version 1.2.3, snakePipes’ DNA-mapping routine can handle RELACS data by performing demultiplexing of fastq files on RELACS adaptors and UMI-based deduplication. Together, AutoRELACS and snakePipes build a continuous workflow that automates ChIP-seq data generation and analysis, allowing for unprecedented processivity.

In this work, we test the performance of AutoRELACS by assessing (1) the scalability of the chromatin barcode integration step, (2) the quality of the generated data in comparison to the benchmark set by the manual protocol, and (3) the sensitivity of the automated method when working with low (≤ 25,000 cells/sample) and very low (≤ 5,000 cells/sample) cell numbers. We show that AutoRELACS is a scalable method that can generate high quality ChIP-seq data, comparable with the standards of the manual protocol. We finally show that AutoRELACS provides reliable epigenetic profiling also with limited input biological material.

## Results

### AutoRELACS is a scalable method for the generation and analysis of ultra-parallelized ChIP-seq data

Restriction Enzyme-based Labeling of Chromatin in Situ (RELACS) is a method that enables the high-throughput generation of ChIP-seq experiments^9^. To increase the standardization and the scalability of this approach, we have developed AutoRELACS, an automated implementation of the RELACS protocol using the liquid handler Biomek i7.

**Figure 1:**
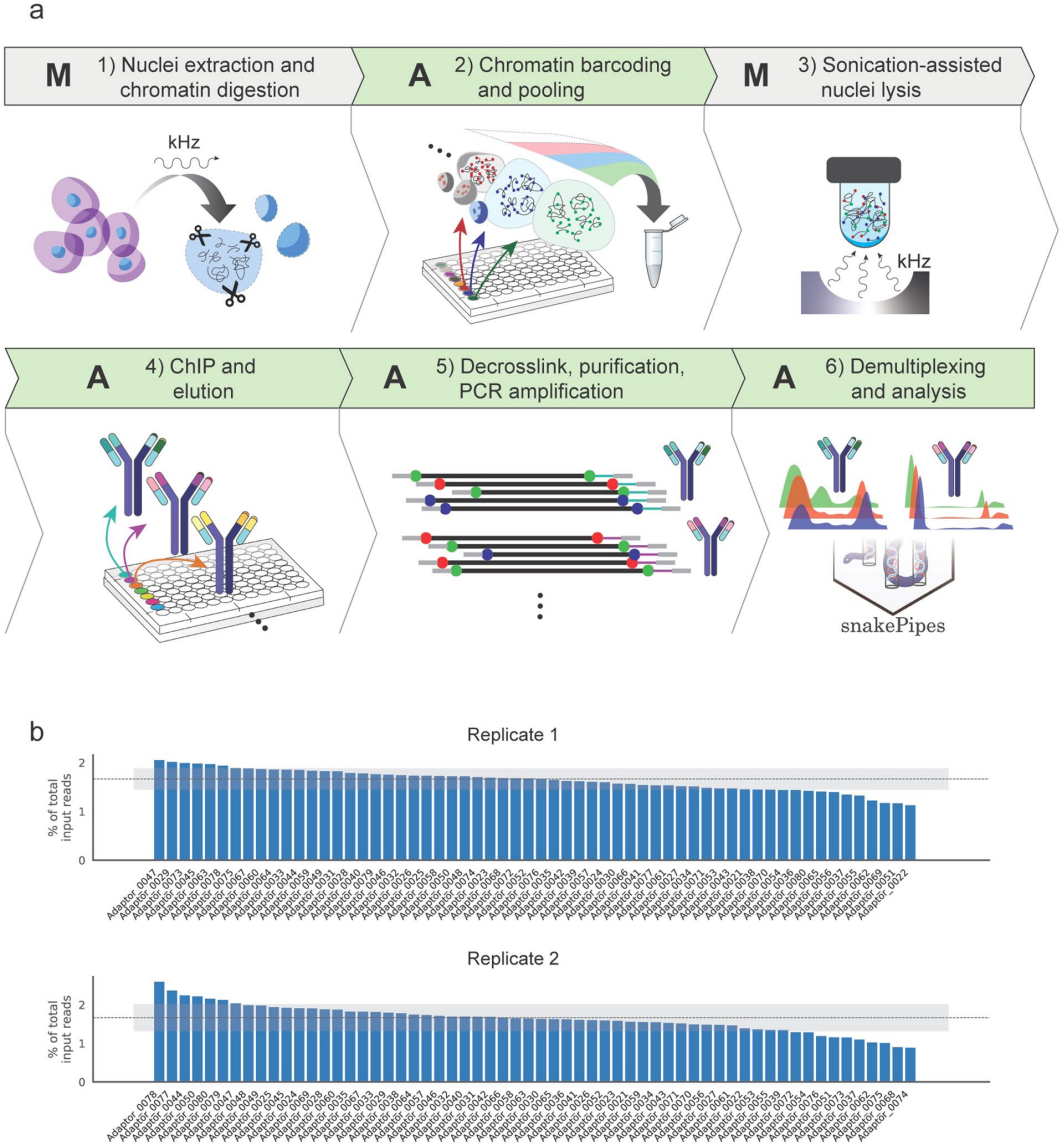
AutoRELACS workflow ensures comprehensive integration of RELACS barcodes. (**a**) Overview of AutoRELACS protocol. **(**1-M) Nuclei of formaldehyde-fixed cells are extracted manually using adjusted ultrasound^14^. The nuclear envelope is permeabilized, and the chromatin digested in situ using a 4-cutter restriction enzyme (RE). (2-A) Digested chromatin from each sample is automatically barcoded. Upon completion, the liquid handler pools all barcoded samples into a unique tube (Biomek i7 program: “RELACS_Barcoding”). (3-M) Pooled samples are collected by the user and nuclei are lysed using focused sonication. (4-A) The barcoded chromatin is aliquoted according to the number of required immunoprecipitation (IP) reactions into corresponding ChIP reaction mixes. The ChIP reactions are carried out overnight in parallel at room temperature on the Biomek i7 workstation. Upon completion, the ChIP-ped chromatin is sequestrated using beads and automatically washed 4 times at increasing stringency conditions and finally eluted in the elution buffer (Biomek program: “RELACS_ChIP_Elution”). (5-A) Subsequently, the eluted chromatin is decrosslinked and the DNA is purified. DNA is amplified via PCR using primers carrying Illumina dual indexes. Optionally, the liquid handler performs multiple rounds of purification and size selection using Ampure XP beads (Biomek program: “RELACS_Decrosslink_FinalLibraries”). A: Automated; M: Manual. Cells images in step1 were made by Freepick from www.flaticon.com. The image was created using Adobe Inc. (2020). *Adobe Illustrator*. Retrieved from https://adobe.com/products/illustrator. (6-A) Libraries are sequenced on Illumina’s sequencing devices. Upon completion of the sequencing run, bcl2 files are automatically converted to fastq format and input into the fully automated ChIP-seq workflow available as part of the snakePipes suite^13^. SnakePipes’ ChIP-seq workflow performs demultiplexing of reads on RELACS custom barcodes, quality controls, mapping and filtering of duplicate reads using unique molecular identifiers (UMI), and further downstream analysis like generation of input-normalized coverage tracks and peak calling. (**b**) Distribution of RELACS barcodes in two independent input chromatin pools. 60 barcodes are integrated into the digested chromatin of two independent batches of S2 cells. Sequencing of the input chromatin pool for replicate 1 (upper panel) and replicate 2 (lower panel), reveals the percentage of input reads for each barcode used (y-axis). The ideal uniform distribution (100/60) is represented as a dotted line. The shaded gray area shows one standard deviation from the mean of the observed distribution.

The AutoRELACS workflow is conceptually divided in six parts: four fully automated (A) processes intermitted by two manual (M) steps (Fig. 1a). First, cells are manually processed to isolate the nuclei^14^ and to digest the chromatin within the nuclear envelope (step 1-M). Next, using the liquid handler Biomek i7, the chromatin from each sample is barcoded and pooled into a unique masterbatch (step 2-A). Using focused sonication, nuclei are lysed and the barcoded chromatin is released (step 3-M). The final three steps of the protocol have been fully automated and require minimal human supervision. These include the chromatin immunoprecipitation (ChIP) reactions and washing steps of beads-bound immunocomplexes (step 4-A), decrosslinking, DNA purification and PCR amplification (step 5-A) and, after sequencing, barcode demultiplexing and bioinformatics analysis with snakePipes (step 6-A)^13^.

The integration of sample-specific RELACS barcodes into the digested chromatin (Fig. 1a, step 2) is key to the success of the method. To test the performance of automated and parallelized RELACS barcode integration, 60 custom barcodes were designed, each composed of a 4 nucleotide (nt)-long unique molecular identifier (UMI), followed by a 8 nt-long barcode with 50% GC content (note that after combining forward and reverse reads, each fragment is tagged by a 8-nt long UMI). These adaptors were used to label the chromatin of 60 batches of S2 cells (*Drosophila melanogaster*) in duplicates using the Biomek i7 workstation (Fig. 1b). Results show that all barcodes are present within the pooled chromatin in both replicates, with a distribution of barcode representation equal to 1.64% ± 0.22% and 1.64% ± 0.35% for replicate 1 and 2 respectively, close to the uniform expectation of 1.667% (Fig. 1b, dashed line).

In summary, we show that AutoRELACS can be used to uniformly integrate multiple barcodes in a fully automated fashion, allowing for ultra-parallelized processing of a considerable number of samples in one single run.

### The quality of AutoRELACS ChIP-seq data is comparable with manual RELACS

Next, we test the quality of the ChIP-seq data generated with AutoRELACS and we compare it with the results from the previously published manual RELACS protocol. To this end, we run in parallel a manual and an automated RELACS experiment where we digest and barcode 28 batches of S2 cells and we immunoprecipitate against H3K4me3, H3K27ac and H3K27me3.

**Figure 2:**
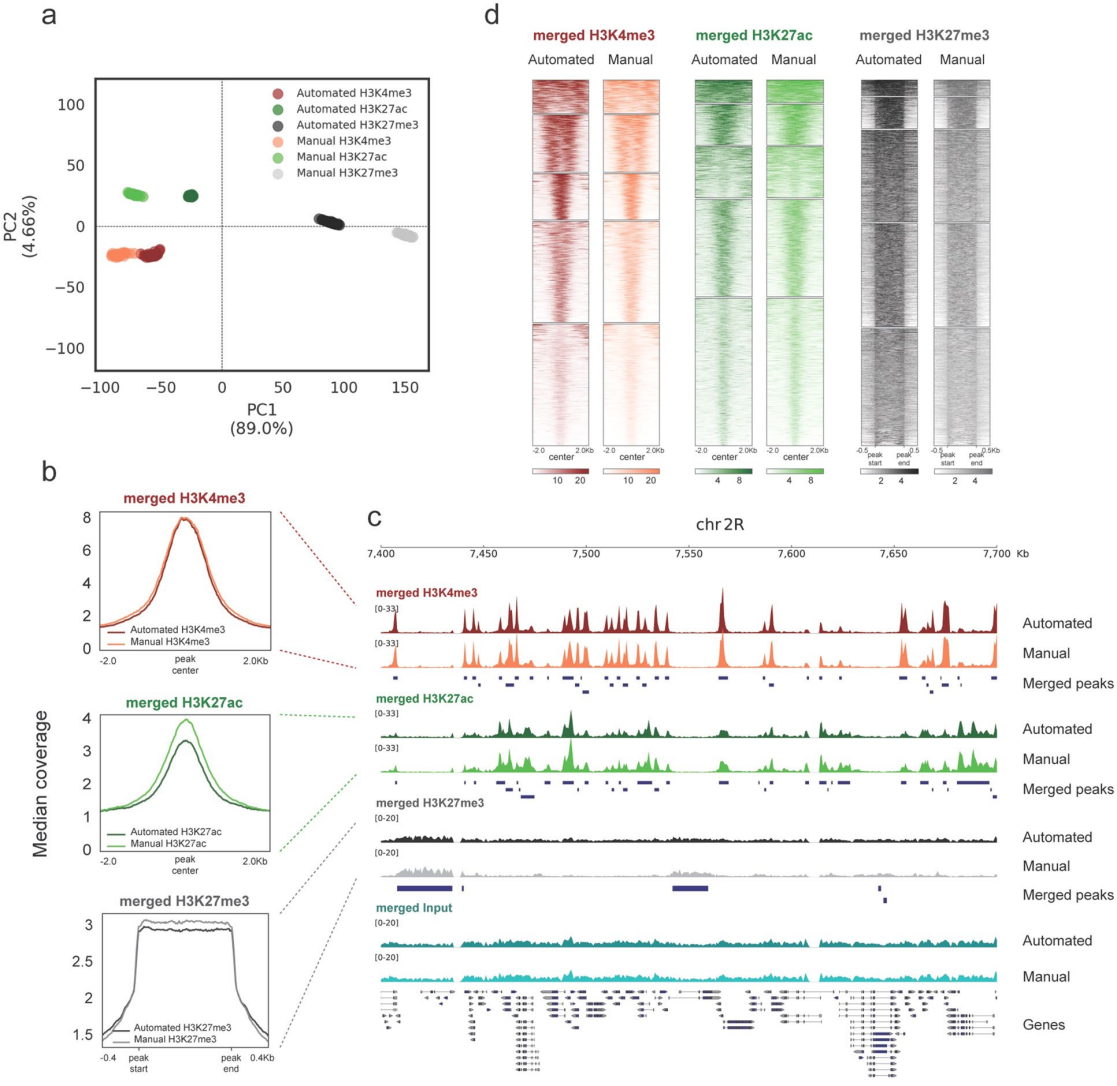
AutoRELACS ChIP-seq data are comparable with the standards of the manual protocol. (**a**) Principal component analysis (PCA) of the normalized coverage matrix computed on the merged peak set between H3K4me3, H3K27ac and H3K27me3, as generated by AutoRELACS (Automated) and manual RELACS (Manual). For each mark and protocol implementation, all 28 demultiplexed technical replicates are shown. The 10,000 most variable loci across all marks are input into the PCA. (**b**) Metaprofile of the median normalized coverage computed over H3K4me3 (upper panel), H3K27ac (central panel) and H3K27me3 (lower panel) peaks. Each panel shows the signal generated with AutoRELACS and manual RELACS from a merge of all 28 technical replicates. For H3K27ac and H3K27me3, we only observe a slight reduction in the signal-to-noise ratio of the automated method. (**c**) Data tracks of the merged signal of the 28 technical replicates for H3K4me3 (red), H3K27ac (green), H3K27me3 (grey) and Input (cyan) on the dm6 locus chr2R:7,400,000–7,700,000. For each mark, we show the profile generated by AutoRELACS (Automated) and manual RELACS (Manual) and the merged set of peaks called in the two datasets (Merged Peaks). (**d**) Heatmaps showing the clustered signal (k = 5) on a merged set of peaks, as identified in the AutoRELACS (Automated) and in the manual RELACS (Manual) dataset, for H3K4me3 (left panel), H3K27ac (central panel) and H3K27me3 (right panel). The choice of colors is the same as in (**a**–**c**) and the similarity between each pair of samples indicates that there are no obvious implementation-specific clusters.

The histone modification profiles generated with manual RELACS and with AutoRELACS are overall similar. The variance present in the first two principal components of the normalized coverage matrix (computed on the merged peaks set) discriminates between the three histone modifications, regardless of the method used (Fig. 2a). Comparison of the metaprofiles of the merged scores over peaks shows identical signal for H3K4me3, while H3K27ac and H3K27me3 present a slightly lower median coverage in AutoRELACS compared to the manual procedure (Fig. 2b). Nevertheless, these differences do not impinge on the sensitivity of the assay. Visual inspection of the normalized coverage reveals high similarity between the two RELACS implementations (Fig. 2c), and high pairwise correlation between demultiplexed samples (Supplementary Fig. 1).

To provide a global overview for all enriched regions, we cluster (k = 5) the signal of H3K4me3, H3K27ac and H3K27me3 using the manual and the automated RELACS data on a common merged peaks set (Fig. 2d). We do not observe any set of peaks that are specific to manual RELACS or AutoRELACS, which shows no obvious implementation-specific biases.

Together, we show that AutoRELACS yields high quality ChIP-seq data that are overall comparable with the manual RELACS protocol.

### AutoRELACS works reliably with low cell numbers

**Figure 3:**
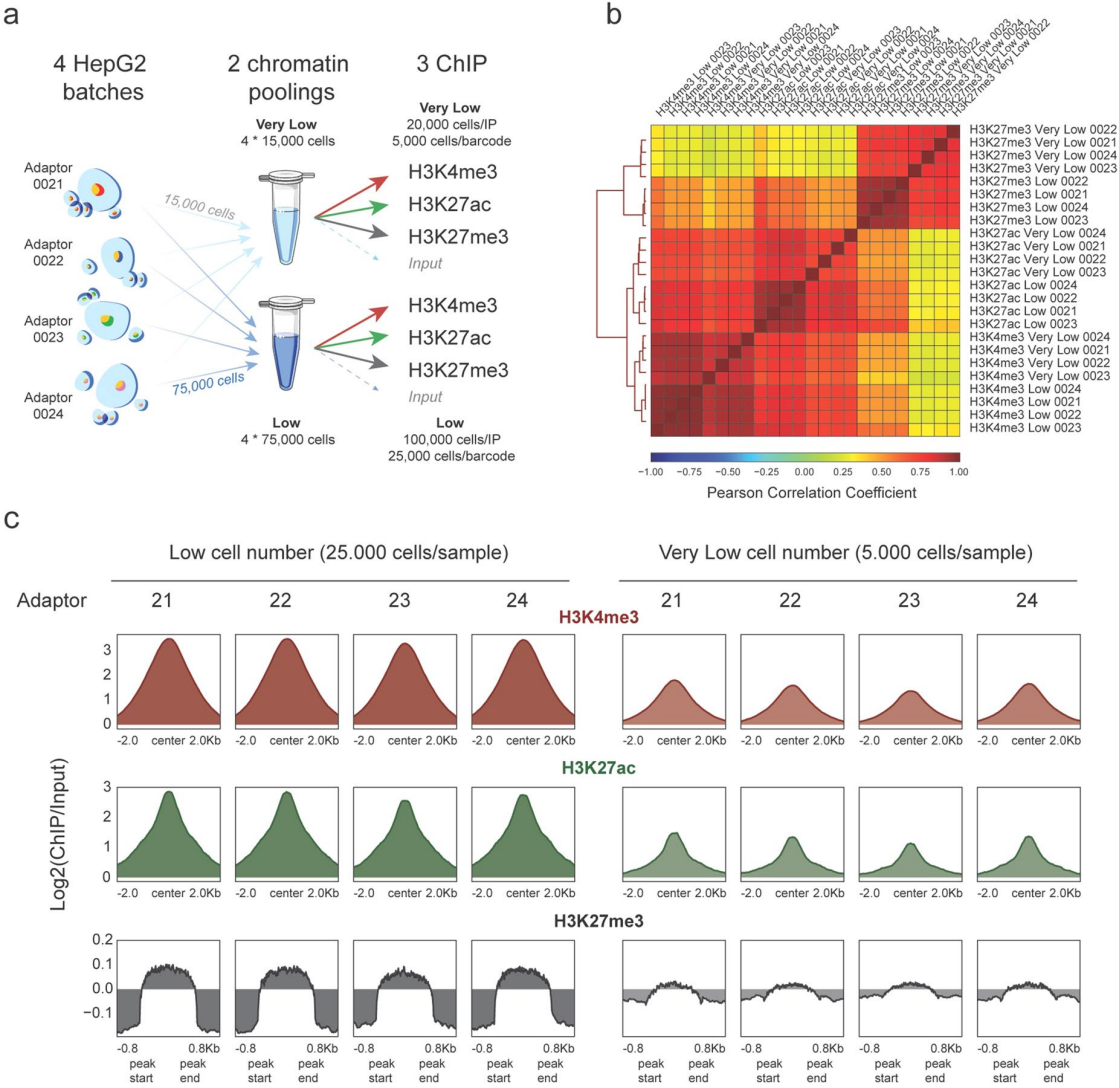
AutoRELACS works with low cell numbers. (**a**) Overview of the experimental design used to test the sensitivity limits of AutoRELACS. Four batches of HepG2 cells are barcoded and pooled into two chromatin masterbatches, the first comprising 4 × 15,000 cells (Very Low input) and the second 4 × 75,000 cells (Low Input). Each chromatin pool is evenly split into three ChIP reactions (H3K4me3, H3K27ac, H3K27me3), while a small fraction (~ 1 μl) is set aside as Input control. For the Very Low pool, about 20,000 cells are used in each ChIP, which corresponds to 5,000 cells/barcode. For the Low pool, about 100,000 cells are used in each ChIP, which corresponds to 25,000 cells/barcode. Cells images were adapted from an flaticon by Freepick from www.flaticon.com. The image was created using Adobe Inc. (2020). *Adobe Illustrator*. Retrieved from https://adobe.com/products/illustrator. (**b**) Hierarchical clustering of HepG2 ChIP-Seq profiles of H3K4me3, H3K27ac and H3K27me3, generated using Low and Very Low chromatin input, based on the pairwise Pearson Correlation Coefficient (PCC). Each pairwise PCC is computed based on the binned coverage (bin width = 10 kb) over the whole genome. (**c**) Metaprofile of the mean enrichment over Input of H3K4me3 (upper panel, red), H3K27ac (central panel, green) and H3K27me3 (lower panel, grey), computed on a consensus set of peaks identified for each mark separately, from the Low and Very Low input chromatin.

RELACS can generate robust epigenetic profiling with low cell numbers^9^. To test the sensitivity limits of AutoRELACS, we barcode 4 batches of HepG2 cells and we aliquote the chromatin into two pools containing 4 × 15,000 and 4 × 75,000 cells respectively. We name the former “Very Low” and the latter “Low” chromatin pool. Next, we divide each chromatin pool into three equal aliquotes for immunoprecipitation against H3K4me3, H3K27ac and H3K27me3, while a small fraction of each pool (~ 1 μL) is set aside as Input control. This setup results in three ChIP reactions with 5,000 cells/barcode for the “Very Low” pool and three ChIP reactions with 25,000 cells/barcode for the “Low” pool (Fig. 3a).

The normalized genome-wide coverages coming from Low and Very Low experiments are highly correlated within histone modifications groups, which indicates that the profiles generated with different amounts of input material are overall similar (Fig. 3b). Although we observe a deterioration of the signal-to-noise ratio in the Very Low group, the enrichment is preserved and, for narrow euchromatic marks, this is sufficient for robust peak calling (Fig. 3c).

In summary, we show that AutoRELACS can be deployed for automated and parallelized profiling of histone modifications genome-wide also for limited amounts of biological samples.

## Discussion

In this work we present AutoRELACS, an automated implementation of the RELACS protocol^9^ that enables the automated high-throughput generation of ChIP-seq experiments. AutoRELACS natively interfaces with the computational pipelines offered by snakePipes^13^, thus streamlining the generation and analysis of histone modifications profiles at unprecedented scale.

RELACS can parallelize ChIP-seq data generation through in situ ligation of sample-specific barcodes into the digested chromatin inside the nuclear envelope. Here, we show that AutoRELACS successfully integrates a high number of barcodes in parallel, ensuring a balanced representation of each adaptor in the final chromatin pool. While we limit our test to 60 barcodes, a single AutoRELACS experiment can support the integration of up to 96 barcodes. The resulting chromatin pool can be split into 96 ChIP reactions, leading to the generation of up to 9,216 independent chromatin profiles in only 3 days. It should be noted that more imbalanced barcode distributions within the final chromatin pool may still lead to a successful profiling, at the cost of increasing the total sequencing depth. It is therefore suggested to perform a preliminary shallow sequencing of the chromatin input to estimate the total sequencing depth needed to ensure a minimum coverage for all samples.

In our implementation we have used the Biomek i7 platform, but the protocol could be equally implemented on any liquid handler that has an on-deck thermocycler, can handle magnetic beads and is able to work on multiple samples in parallel (for further details, we refer to the Supplementary Informations: Biomek i7 requirements and consumables for automation).

AutoRELACS carries all advantages and limitations of RELACS. As with all automated protocols, the large degree of standardization offered by AutoRELACS comes with reduced flexibility, which might be required for individual samples in one batch. AutoRELACS defines batch-wide parameters (e.g. ChIP volumes, beads volume) that can be adjusted by the user. Samples requiring distinct treatment should be processed in different batches.

The current AutoRELACS implementation has room for further improvements. To date, the method still requires human intervention in the earliest stages of the protocol. Future developments might integrate the use of focused sonicator platforms into the workflow of the liquid handler workstation, to further reduce user intervention and enable a full walk-away automated solution. It should be noticed that our method, in contrast to other sensitive ChIP-seq protocols^5^ employs a standard library preparation. We consider this an advantage, but in future work one might want to combine improvements at both the sample preparation and the library preparation steps.

Although in this work we have focussed on histone modifications, there is no fundamental limitation to profiling also transcription factor binding sites. Depending on the specific factor or cofactor of interest, this may require improved fixation conditions before the AutoRELACS protocol or variable ChIP conditions in step 4 of the AutoRELACS protocol. Regardless of such optimizations and extensions, the current AutoRELACS protocol defines a common set of standard operating procedures to generate high quality ChIP-seq data, comparable with the standards of the manual implementation. Importantly, the method can be used for epigenetic profiling of low cell numbers. Together, these features suggest AutoRELACS as a method of choice in various clinical applications, potentially enabling comprehensive screening of epigenetic markers from small amounts of biological material.

## Materials and methods

### Cell culture

S2 cells were cultured in Express Five SFM (Thermo Fisher Scientific) supplemented with glutamax, at 27 °C and were provided by Akhtar’s lab (MPI-IE). HepG2 liver hepatocellular carcinoma (ATCC, HB-8065) were cultured in Eagle’s minimal essential medium (EMEM, Lonza, 06-174) supplemented with 10% fetal bovine serum (Sigma), 2 mM l-glutamine (Lonza), 1.8 mM CaCl_2_, 1 mM sodium pyruvate (Lonza) and penicillin–streptomycin mixture (100 units/mL, Lonza), at 37 °C at 5% CO_2_ in 10 cm plates, up to 70–80% confluency.

### Cell fixation

HepG2 and S2 cells were fixed in 1% methanol-free formaldehyde (Thermo Scientific, 28,906) in D-MEM (for HepG2 cells) or Express Five SFM (for S2 cells) for 15 min at room temperature under gentle shaking. Formaldehyde was quenched for 5 min by adding 125 mM glycine final concentration. Cells were rinsed twice with ice-cold PBS, harvested by scraping (HepG2) and pelleted (300 g, 10 min, 4 °C).

### Detailed AutoRELACS workflow

The AutoRELACS protocol is divided into five main steps (as described in Fig. 1).

A separated program file is provided for each automated section and is available for download at https://github.com/FrancescoFerrari88/AutoRELACS/tree/master/AutoRELACS_binaries_Biomek_i7.

#### 1. Nuclei extraction and chromatin digestion (manual protocol)

Nuclei are extracted from fixed cells, swollen, digested, washed and counted as previously described^9^. The resulting digested nuclei are resuspended in 10 mM Tris–HCl pH 8 at the nuclei density of 500,000 nuclei/25 µL (Drosophila S2) and 500,000 nuclei/25 µL (HepG2) for the following nuclei barcoding step.

#### 2. Chromatin barcoding and pooling (automated, method file “RELACS barcoding.bmf”)

In this step chromatin is barcoded inside the nuclei as previously described^9^, but using automation. This method allows the processing for a flexible number of nuclei samples, from 1 to 96.

Preparation of reagents: nuclei samples are aliquoted column-wise in a 96-wells PCR plate (25 µL of digested nuclei per well), named “Nuclei Plate”. 2 µL of the desired RELACS barcode at 15 µM are aliquoted in each well of a second 96-wells PCR plate, following the same coordinates of the respective nuclei aliquot (named “Index Plate”). The following reagent mixes are positioned into 1.5 mL conical tubes on the Biomek deck in a cold Peltier block: End Repair mix (ER), Ligation mix (LIG) and 3 M NaCl, following directions as highlighted in the “guided instrument setup” (a screenshot of the deck is shown in Supplementary Fig. 2a).

Steps of the “RELACS barcoding” program: 5 µL of ER mix are added into each occupied well of “Nuclei Plate”. The plate is mixed on the orbital shaker present on the deck and incubated into the integrated PCR cycler for 30 min at 20 °C and for 5 min at 65 °C. End-repaired nuclei are transferred from “Nuclei Plate” to the “Index Plate” containing RELACS barcodes. 15.5 µL of LIG mix are added into each occupied well. The “IndexPlate” is shaken and transferred into the integrated PCR cycler for ligation incubation (15 min at 30 °C and for 15 min at 20 °C). The ligation is inactivated adding 5 µL of 3 M NaCl into each occupied well of “Index Plate”. The plate is shaken and pooling is automatically performed by transferring samples from each occupied well of “Index Plate” to 1.5 mL tubes positioned into the “Final Pool” rack. Wells containing barcoded nuclei can be pooled as specified by the user, by indicating source and destination coordinates of “Index Plate” and “Final Pool” into the .csv file “Nuclei_Pooling_Template.csv”.

#### 3. Sonication-assisted nuclei lysis (manual protocol)

Tubes containing nuclei pools are manually collected. Barcoded nuclei are pelleted down (5,000 g for 10 min). Supernatants are discarded and pellets are resuspended into the desired volume of Shearing buffer supplemented with Protease Inhibitor Cocktail (Roche, 11873580001) and sonicated for 5 min in a Covaris E220 sonicator as described^9^.

#### 4. ChIP and elution (automated, method file “RELACS ChIP-Elution.bmf”)

The method allows for a flexible number of ChIP reactions from 1 to 96 simultaneously. A screenshot of the overall organization of the deck is shown in Supplementary Fig. 2b.

All reagents used and the procedure of ChIP largely overlap to the ones described in our former publication^9^, with the relevant modifications highlighted here below. Preparation of ChIP plate (named “Sample Plate”): ChIP reactions are carried out in a maximum volume of 150 µL instead of 200 µL used for manual RELACS. 75 µL of chromatin prepared in step 3 are aliquoted column-wise into a 1.2 mL storage plate (Thermo Fisher, AB1127) accordingly to the required number of ChIP. To equilibrate salts and detergents, 73 µL of 1X buffer iC1 (from iDeal ChIP-seq kit for histones, Diagenode C01010173) supplemented with Protease Inhibitor Cocktail (Roche, 11873580001) and 2 µL of 5 M NaCl are added into each chromatin well. One µg per 100,000 cells of the desired antibody (H3K4me3 C15410003, H3K27ac C15410196, H3K27me3 C15410195, all from Diagenode) is added into each well. Remaining chromatins are set aside at 4 °C to prepare inputs. Please notice that input samples will be manually added later on before the automated decrosslinking step.

Preparation of reagents: ChIP Wash buffers 1 to 4 (from iDeal ChIP-seq kit for histones, Diagenode C01010173) are aliquoted into quarter module reservoirs divided by length. ChIP elution buffer (1% SDS, 200 mM NaCl, 10 mM Tris–HCl pH 8, 1 mM EDTA) is also aliquoted into the remaining well of the reservoir as highlighted in the “guided instrument setup”. ChIP beads (Dynabeads protein A-conjugated magnetic beads, Invitrogen) are washed twice with 1X buffer iC1 and aliquoted into two 1.5 mL conical tubes before placing them on the deck.

Automated protocol: the program involves four main steps (antibody incubation, beads incubation, ChIP washes, elution). Antibody incubation is performed by shaking the “Sample Plate” containing the ChIP reactions on the orbital shaker, repeating this procedure 12 times: 20 min continuous shaking at 500 rpm, stop for 10 min. In comparison to manual RELACS we carried out ChIP incubation for a total time of 6 h at room temperature instead of 10 h at 4 °C as used in manual RELACS. Please notice that we did this modification to overcome technical constraints that would have resulted in loss of samples when mixing by pipetting.

Beads incubation: beads placed on the deck are automatically mixed and 15 µL of beads are dispensed into each ChIP reaction. “Sample Plate” is then transferred on the orbital shaker and mixed for a total time of 2 h at room temperature (5 min continuous shaking at 500 rpm, stop for 5 min, repeated 12 times). In comparison to the procedure used for manual RELACS, beads incubation time for AutoRELACS has been reduced by one hour.

ChIP washes: the following procedure is repeated for each of the four wash buffers. “Sample Plate” is transferred onto the magnetic rack and left for 5 min to reclaim the beads-bound immunocomplexes to the magnet. Supernatants are aspirated, discarded into the wash station, and 150 µL of wash buffer are added into each occupied well. Plate is shaken on the orbital shaker for about 5 min to wash the beads (5 s pulse shaking at 800 rpm for 60 times).

Elution: the last wash supernatants are removed from the beads. 80 µL of ChIP elution buffer is added to the beads and the plate is shaken on the orbital shaker for a total time of about 35 min (5 s pulse shaking at 800 rpm for 60 times, 4 min pause, for four times). “SamplePlate” is placed onto the magnet for 5 min and supernatants containing immunoprecipitated material are collected into a fresh 96-well plate (called “ChIPEluates”) and stored overnight into the integrated PCR cycler at 10 °C.

#### 5. Decrosslink, purification, USER treatment, PCR amplification (automated, method file “RELACS Decrosslink-FinalLibrary.bmf”)

The plate “ChIP Eluates” is collected from the Biomek and Input samples are manually added column-wise after the ChIP samples (0.1–10% of the original chromatin volume in 80 µL of ChIP Elution buffer). This plate is placed back onto the deck and renamed in the instrument setup as “Sample Plate 2”.

Reagent preparation: 4 µL of 10 µM Illumina dual index primer cocktails (from IDT) are placed in a 96-well PCR plate column-wise following the desired pattern corresponding to the ChIP samples (plate is named “Index Plate”). The following reagents are required for this section of program, as specified in the instrument setup (Supplementary Fig. 2c): 100% isopropanol, EB (10 mM Tris–HCl pH 8), freshly prepared 85% ethanol (all on the deck at room temperature), proteinase K 20 mg/ml (Thermo Fisher, EO0491), glycogen 20 mg/mg (Thermo Fisher, R0561), carboxylated magnetic beads (Invitrogen, 65011), PCR mix (NEBNext Ultra II Q5 Master mix, NEB M0544), USER enzyme (NEB M5505), all placed in 1.5 mL conical tubes in a cold Peltier block. Ampure XP (Beckman Coulter, A63881) are thoroughly mixed and aliquoted column-wise according to the pattern of “Sample Plate 2” in a 96-well storage plate (AB0765, Thermo Fisher), using 100 µL of beads per well.

Automated Decrosslink: 2 µL of proteinase K are transferred into each occupied well of “Sample Plate 2” containing ChIP eluates and input samples. The plate is mixed on the orbital shaker and incubated for 2 h at 65 °C into the integrated PCR cycler.

Automated DNA purification: in comparison to manual RELACS, in which decrosslinked DNA is purified using columns (Qiagen minElute PCR purification kit), AutoRELACS uses a custom-made DNA purification by precipitation and sequestration using carboxylated magnetic beads. Decrosslinked samples are transferred from the PCR plate to a larger 96-well storage plate (“ChIP Purification”, 4titude, LB0125). The following reagents are added into each occupied well: 2 µL of glycogen, 10 µL of carboxylated beads (automatically pre-mixed by pipetting before dispensing), and 80 µL of isopropanol. The plate “ChIP Purification” is mixed by shaking and incubated at room temperature for 10 min. The beads are reclaimed onto the integrated magnet for 5 min and supernatants are discarded. DNA bound to beads is washed twice using 200 µL of 85% ethanol. Beads are dried and DNA is automatically eluted by addition of 28 µL of EB into each occupied well. Plate is placed onto the magnet to discard the beads and to collect purified eluates.

USER treatment: 27 µL of purified DNAs are collected into a fresh 96-well PCR plate. 3 µL of USER enzyme is added into each occupied well. Plate is shaken and incubated into the integrated PCR cycler for 15 min at 37 °C. Samples are transferred into a 96-well storage plate for purification using Ampure XP (0.9X ratio). After purification, samples are eluted in 22 µL of EB.

Automated amplification of final libraries and purification: 21 µL of each purified DNA are transferred to the 96-well PCR plate containing Illumina indexes (“Index Plate”). 25 µL of PCR mix are added into each occupied well and the plate is shaken. The plate is then transferred into the integrated PCR cycler for PCR incubation (hot start 98 °C for 30 s; PCR cycles: 98 °C for 10 s, 65 °C for 75 s; final extension 65 °C for 5 min). Notice that before launching the method the user has the possibility of choosing the number of PCR cycles to use (10, 12 or 14). In the experiments presented in this work libraries were amplified using 12 PCR cycles (14 PCR cycles for low input ChIP). Amplified samples are transferred into a 96-well storage plate for double purification using Ampure XP (first at 0.8X ratio second at 1X ratio). Ready libraries are eluted in 25 µL of EB and transferred in a clean 96-well PCR plate.

### Sequencing

Libraries were quality-controlled to check the concentration (Qubit DNA HS, Invitrogen, Q32851) and the fragment size distribution (Fragment Analyzer capillary electrophoresis, NGS 1–6,000 bp hs DNA kit). Libraries were pooled and normalized to 1 to 2 nM with 10% PhiX spike-in according to the Illumina guidelines. Libraries were clustered on NovaSeq XP flowcells and sequenced paired-end with a read length of 50 bp on an Illumina NovaSeq 6000 instrument.

### Bioinformatics analysis

BCL files were converted to fastq format using bcl2fastq2 (v. 2.20.0) and demultiplexed on Illumina barcodes. Fastq files were used as input to snakePipes’ DNA-mapping and ChIP-seq workflows (v. 1.2.3)^13^, using default parameters as listed in https://github.com/FrancescoFerrari88/AutoRELACS/tree/master/snakePipes_defaults. The only added requirement was the provision of a sample sheet, an example of which is provided in the same repository. Mapping was performed on the genome build dm6 and hg38 for *D. melanogaster* and *H. sapiens* respectively. Briefly, fastq files were demultiplexed on RELACS adaptor barcodes and reads were mapped to the reference genome using Bowtie2 (v. 2.3)^15^. Uniquely mapping read pairs (mapq > 3) were retained and duplicates were filtered on UMI using UMITools (paired mode) (v. 1.0.0)^16^. Peaks were called using MACS2 (v. 2.1.2)^17^ with default parameters. Merged peak sets were obtained by concatenating, sorting and merging peaks identified in the different experimental conditions included in the analysis, using bedtools sort | merge (v. 2.28)^18^.

Clustered heatmaps, ChIP-seq metaprofiles and the clustered correlations heatmap were generated using deeptools (v. 3.3.1)^19^, using filtered bam files as input. Principal component analysis (Fig. 2a) was performed using the Python library scikit-learn (v. 0.19.1) on rlog-transformed count matrix^20^. Coverage was obtained using deeptools’ multiBamSummary (v. 3.3.1)^19^ on the merged peak set. We use pyGenomeTracks^21^ to visualize signal tracks on specific genomic loci.

## Supplementary information

Supplementary Legends.

Supplementary Figure 1.

Supplementary Figure 2.

## Data Availability

Raw data and normalized bigWig tracks were deposited to GEO and are available for download using the following accession number: GSE147042.

## Code availability

The fully reproducible and documented analysis is available on github at https://github.com/FrancescoFerrari88/AutoRELACS, as Jupyter notebooks and R/python scripts.
