# Supplementary material for: AutoRELACS: automated generation and analysis of ultra-parallel ChIP-seq
Source: Sci Rep. 2020 Jul 24;10:12400. doi: 10.1038/s41598-020-69443-8 (PMC7381599; doi:10.1038/s41598-020-69443-8)
Supplement: Supplementary file 1 — Supplementary Legends. [file 41598_2020_69443_MOESM1_ESM.docx]

**SUPPLEMENTARY**

**Biomek i7 requirements and consumables for automation**

The following instrument parts are required to perform AutoRELACS on the Biomek i7: Biomek i7 Workstation equipped with left and right pods; 1200 µl 96-multichannel (left pod), Span-8 pipets coupled with 1 ml syringe volume (right pod), gripper tools (one per pod), static Peltier with tube block for conical tubes, shaking Peltier and block for 96-well PCR plates, Orbital shaker, Wash station for multichannel, Wash station for Span-8, Magnet, Peristaltic pump (Masterflex L/S, Cole-Parmer), Automated PCR cycler (Thermo Fisher), seven Tip Loading Stations, twenty-six Automated Labware Positioners. Deck configuration details are indicated into each respective protocol part (Supplementary Figure 2). Instrument configuration file is provided in the supplementary material (Biomeki7.bif).

The following plastic consumables are used for automation: Hard-Shell 96-well PCR plates (HSP9601, Bio-Rad), 96-Deep well storage microplates (4titude, LB0125), Low profile 1.2 ml square storage plate (AB1127, Thermo Fisher), 0.8 ml 96-well storage plate (AB0765, Thermo Fisher), Auto-sealing plate lids (MSL2022, Bio-Rad), Universal microplate lid (4ti-0290, 4titude), 300 ml reservoir (EK-2035, Agilent technologies), Modular reservoir quarter module divided by length (372788, Beckman Coulter), Modular reservoir quarter module (372790, Beckman Coulter), sterile tips with filter (all from Beckman Coulter): 1025 µl (B85955), 190 µl (B85911), 50 µl (B85888).

**Supplementary Figure 1: demultiplexed samples from manual RELACS and AutoRELACS are highly correlated**

Heatmap showing the pairwise Pearson correlation coefficient between the demultiplexed samples for H3K4me3 (top-left), H3K27ac (top-right) and H3K27me3 (bottom).

**Supplementary Figure 2: Biomek i7 deck configurations for AutoRELACS.**

a) Deck configuration for the method “RELACS barcoding”. On the deck are present filtered tips in different volumes (50 µl violet box in position 1, 190 µl green boxes in position 4 and 5), PCR lid for automation (3), magnet (2) and Peltier block containing barcoding reagents at 4 °C (4 °C reagents, 6). Digested nuclei are aliquoted in a 96-well PCR plate (Nuclei plate, 7). RELACS barcodes are aliquoted in a 96-well PCR plate (Index plate, 10) positioned on top of a cold Peltier. To protect the indexes, a plastic lid is positioned on top of the plate.

b) Deck configuration for the method “RELACS ChIP-Elution”. On the deck are present filtered tips in different volumes (190 µl green boxes in position 3, 4 and 5, 1025 µl orange box in position 6), PCR lid for automation (2), magnet (1) and Peltier block containing ChIP reagents at 4 °C (4 °C reagents, 8). Room temperature ChIP reagents are stored in reservoirs (ChIP-Wash reagents, 9). ChIP reactions are aliquoted in a 96-deep well storage plate (Sample plate, 10). Final ChIP eluates are transferred into a 96-well PCR plate (ChIP eluates, 7).

c) Deck configuration for the method “RELACS Decrosslink-FinalLibrary”. On the deck are present filtered tips in different volumes (50 µl violet boxes in position 1 8, 9, 190 µl green boxes in position 4, 5, 7, 1025 µl orange boxes in position 6, 10), PCR lid for automation (3), magnet (2) and Peltier block containing the required reagents at 4 °C (4 °C reagents, 18). Room temperature reagents are stored in reservoirs (DNA purification reagents, 23). 85% Ethanol is stored in a lidded reservoir (20). Ampure XP are aliquoted in a 96-deep well storage plate covered with a lid (Ampure XP, 22). ChIP and PCR purification occur in 96-deep well plates (11, 15). 96-well PCR plates in position 12, 16 and 17 are required for several steps of the method and to store the final libraries. ChIP and Input samples, which need to be firstly decrosslinked, are positioned in a 96-well PCR plate (24). The image was created using Adobe Inc. (2020). *Adobe Illustrator*. Retrieved from https://adobe.com/products/illustrator.
