# Supplementary figures and images for: AutoRELACS: automated generation and analysis of ultra-parallel ChIP-seq
Source: Sci Rep. 2020 Jul 24;10:12400. doi: 10.1038/s41598-020-69443-8 (PMC7381599; doi:10.1038/s41598-020-69443-8)

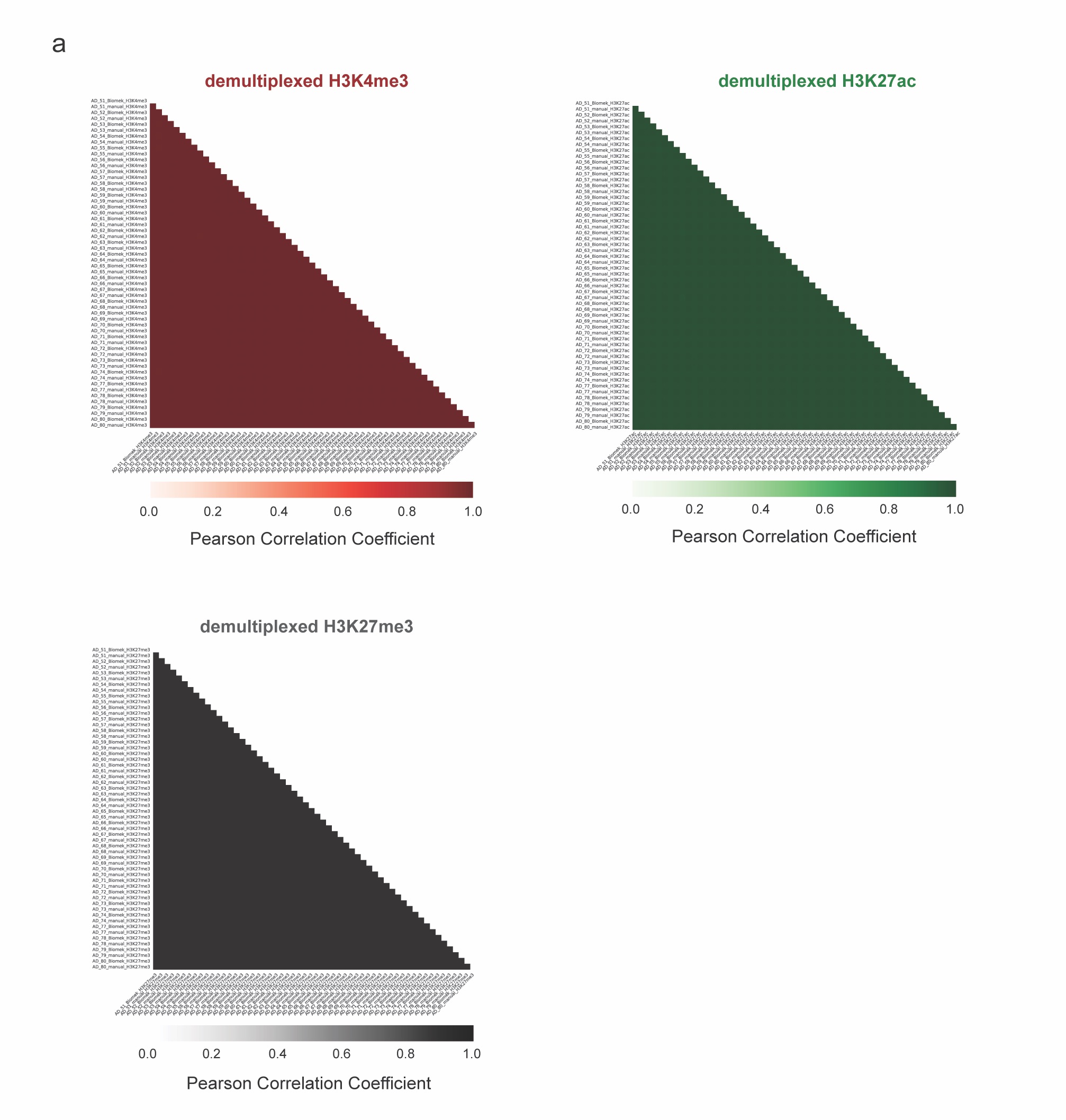

Supplement: Supplementary file 2 — Supplementary Figure 1. [file 41598_2020_69443_MOESM2_ESM.jpg]

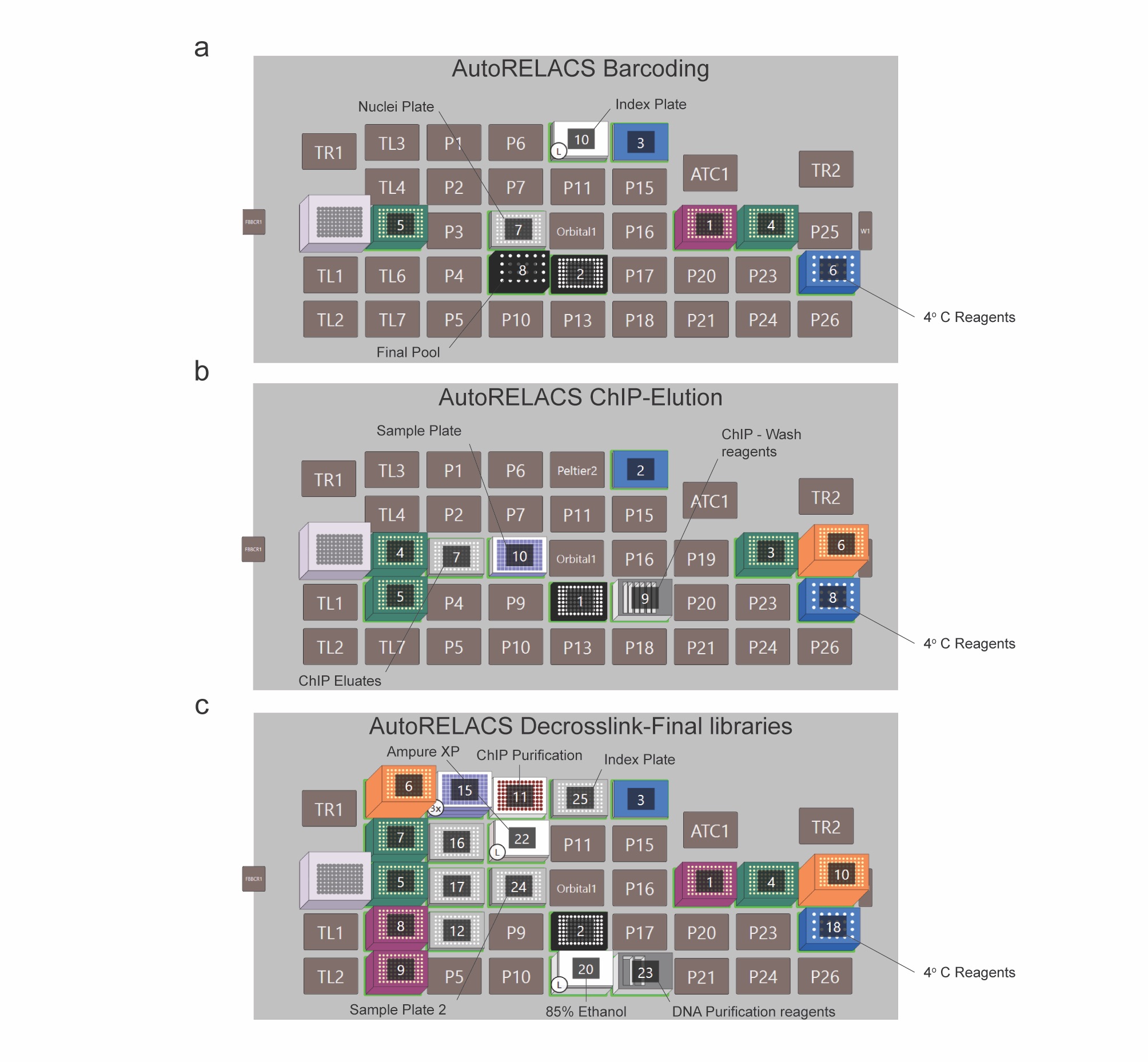

Supplement: Supplementary file 3 — Supplementary Figure 2. [file 41598_2020_69443_MOESM3_ESM.jpg]
